# New 19-Oxygenated Steroids from the Soft Coral *Nephthea chabrolii*

**DOI:** 10.3390/md10061288

**Published:** 2012-06-06

**Authors:** Shang-Kwei Wang, Shyh-Yueh Puu, Chang-Yih Duh

**Affiliations:** 1 Asia-Pacific Ocean Research Center, National Sun Yat-Sen University, Kaohsiung 804, Taiwan; Email: skwang@cc.kmu.edu.tw; 2 Department of Microbiology, Kaohsiung Medical University, Kaohsiung 807, Taiwan; 3 Department of Marine Biotechnology and Resources, National Sun Yat-Sen University, Kaohsiung 804, Taiwan; Email: lh89101kimo@yahoo.com.tw

**Keywords:** *Nephthea chabrolii*, 19-oxygenated steroids, cytotoxicity, anti-HCMV

## Abstract

In order to search for novel bioactive substances from marine organisms, we investigated the acetone extract of the soft coral *Nephthea chabrolii* collected at San-Hsian-Tai, Taitong County, Taiwan. From this extract three new 19-oxygenated steroids, nebrosteroids N–P (**1**–**3**) were isolated. The structures of these compounds were elucidated by extensive spectroscopic analyses.

## 1. Introduction

**Figure 1:**
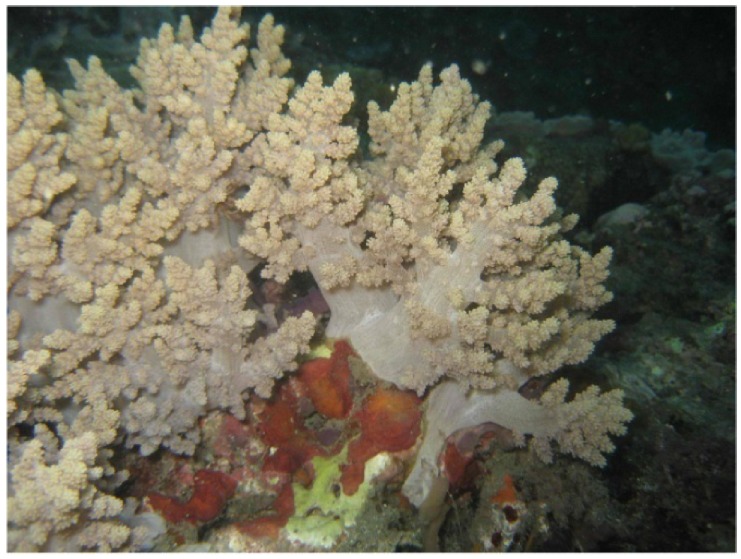
Soft coral *Nephthea chabrolii*.

**Figure 2:**
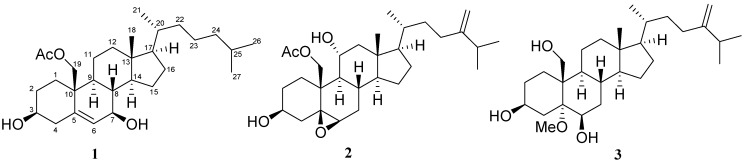
Structures of compounds **1**–**3**.

Numerous secondary metabolites including sesquiterpenoids, diterpenoids, meroditerpenoids, and steroids have been isolated from soft corals of the genus *Nephthea* [1,2,3,4,5,6,7,8,9,10,11,12,13,14,15,16,17,18,19,20,21,22,23,24,25,26]. Previous bioassay results on these materials showed them to exhibit diverse biological properties including cytotoxic [4,5,6,7,18,20], anti-inflammatory [13,14,23,26] and antimicrobial activities [19]. The acetone extract of the soft coral *Nephthea chabrolii* (Figure 1) collected off the San-Hsian-Tai coast, Taiwan, in July 2008 was found to be cytotoxic towards P-388 mouse lymphocytic leukemia cell lines. Chromatographic fractionation led to the isolation of three new compounds, nebrosteroids N–P (**1**–**3**) (Figure 2).

## 2. Results and Discussion

**Figure 3:**
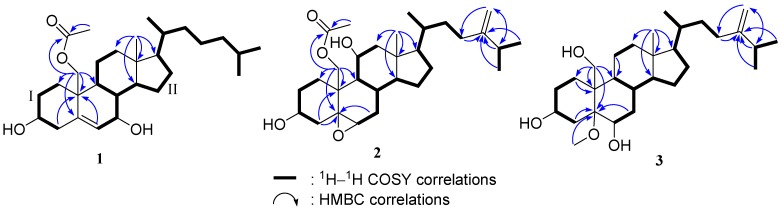
COSY and HMBC correlations of compounds **1**–**3**.

**Figure 4:**
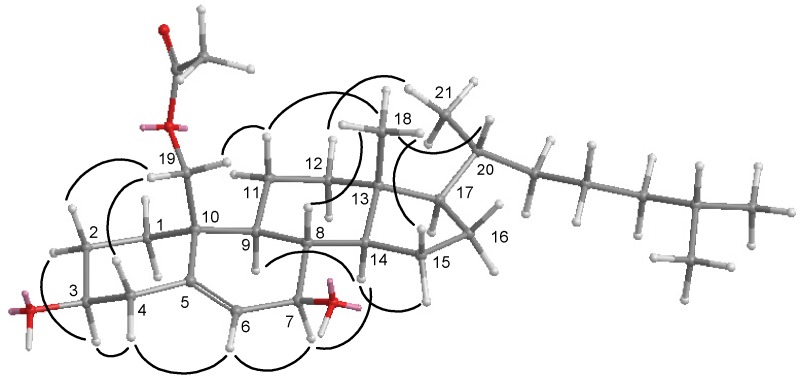
NOESY correlations of compound **1**.

Nebrosteroid N (**1**) had a molecular formula of C_29_H_48_O_4_ as established by interpretation of its HRESIMS and NMR data. The IR spectrum of **1** indicated the presence of hydroxyl(s) (ν_max_3393 cm^−1^) and an ester group (ν_max_ 1738 cm^−1^). Further, the ^1^H NMR spectrum revealed the presence of a tertiary methyl (δ_H_ 0.71), three secondary methyls (δ_H_ 0.92, 0.87, and 0.86), and two oxymethines [δ_H_ 3.59 (1H, m), 3.82 (1H, d, *J* = 7.6 Hz)], and an oxymethylene [δ_H_ 3.99, 4.50 (*J*_AB_ = 11.8 Hz)]. The presence of a trisubstituted double bond was revealed by NMR signals [δ_H_ 5.54 (1H, br s), δ_C_ 129.6 (CH), 138.1 (C_q_)] (Table 1). NMR data of **1** exhibited the presence of an acetoxyl group [δ_H_ 2.07 (3H, s), δ_C_ 21.1 (CH_3_), 170.7 (C_q_)]. The ^13^C NMR and DEPT spectra of **1** contained resonances for eleven sp^3^ methylenes, eight sp^3^ methines, two quaternary sp^3^ carbons, one sp^2^ methine, one quaternary sp^2^ carbon, and one carbonyl. Comparison of NMR chemical shift values of **1** with those of cholest-5-en-3β,7β,19-triol [27] reported from the black coral *Antipathes subpinnata* as well as its HMBC cross-peaks of H_2_-19/C-1,C-5, C-9, C-10, carbonyl carbon at C-19 suggested that **1** may be a 19-acetyl analogue of cholesta-5-en-3β,7β,19-triol. Interpretation of the ^1^H–^1^H COSY spectrum led to partial structures I and II (Figure 3). Rings A and B were elucidated on the basis of HMBC cross-peaks (Figure 3) between H_2_-19/C-1, C-5, C-9, C-10 and H_2_-4, H-6/C-10, whereas rings C and D were completed based on HMBC correlations between H_3_-18/C-12, C-13, C-14, C-17. The NOESY correlations (Figure 4) observed between H-11β and H_3_-18, H-11β and H-19, H-19 and H-4β, H_3_-18 and H-8, H_3_-18 and H-20, H-3 and H-4α, H-6 and H-7, H-9 and H-14, and H-7 and H-14 in **1** confirmed that nebrosteroid N (**1**) was cholesta-5-en-3β,7β,19-triol 19-acetate.

**Table 1:** ^1^H and ^13^C NMR data for compounds **1**–**3** measured in CDCl_3_.

Position	1		2		3	
	δ_H_ *^a^* (*J* in Hz)	δ_C_ *^b^*	δ_H_ *^c^* (*J* in Hz)	δ_C_ *^d^*	δ_H_ *^a^* (*J* in Hz)	δ_C_ *^b^*
1	α: 1.07 m	33.6	α: 1.34 m	35.4	α: 1.72 m	29.4
	β: 2.04 m		β: 2.65 m		β: 1.48 m	
2	α: 1.87 m	31.7	α: 1.80 m	31.4	α: 1.86 m	31.0
	β: 1.44 m		β: 1.34 m		β: 1.33 m	
3	3.59 m	71.1	3.72 m	69.1	3.88 m	67.5
4	α: 2.41 m	41.8	α: 1.40 m	42.7	α: 2.04 m	33.4
	β: 2.31 d (11.6)		β: 2.30 d (12.0)		β: 2.00 m	
5		138.1		61.3		79.3
6	5.54 br s	129.5	2.96 d (2.5)	59.8	3.85 m	69.4
7	3.82 d (7.6)	72.4	α: 1.27 m	31.9	α: 1.52 m	33.4
			β: 2.09 m		β: 1.48 m	
8	1.65 m	41.9	1.70 m	29.6	2.17 m	31.4
9	1.04 m	48.3	0.80 m	57.3	1.42 m	45.0
10		39.5		38.9		43.3
11	α: 1.57 m	21.6	4.08 m	68.9	α: 1.02 m	21.5
	β: 1.47 m				β: 1.46 m	
12	α: 1.12 m	39.7	α: 1.16 m	50.8	α: 1.16 m	40.4
	β: 2.04 m		β: 2.24 m		β: 2.01 m	
13		43.0		43.1		43.2
14	1.07 m	56.6	0.97 m	55.5	1.03 m	57.1
15	α: 1.79 m	26.1	α: 1.59 m	24.0	α: 1.54 m	24.0
	β: 1.42 m		β: 1.07 m		β: 1.03 m	
16	α: 1.87 m	28.5	α: 1.90 m	28.2	α: 1.84 m	28.3
	β: 1.31 m		β: 1.30 m		β: 1.26 m	
17	1.07 m	55.4	1.17 m	55.8	1.06 m	56.0
18	0.71 s	11.9	0.71 s	12.7	0.72 s	12.5
19	3.99 d (11.8)	64.3	4.30 d (12.0)	64.8	3.70 d (12.0)	65.9
	4.50 d (11.8)		4.93 d (12.0)		4.26 d (12.0)	
20	1.37 m	35.7	1.40 m	35.6	1.40 m	35.8
21	0.92 d (6.0)	18.7	0.95 d (6.5)	18.6	0.94 d (6.8)	18.7
22	0.99 m	36.2	1.13 m	34.4	1.14 m	34.7
	1.33 m		1.52 m		1.53 m	
23	1.14 m	23.8	1.87 m	30.9	1.86 m	31.0
	1.32 m		2.09 m		2.10 m	
24	1.12 m	39.5		156.7		156.9
25	1.50 m	28.0	2.22 m	33.8	2.23 m	33.8
26	0.86 d (6.4)	22.8	1.02 d (7.0)	22.0	1.02 d (6.8)	22.0
27	0.87 d (6.4)	22.5	1.03 d (7.0)	21.8	1.03 d (6.8)	21.9
28			4.65 s	106.0	4.66 s	105.9
			4.72 s		4.71 s	
OAc	2.07 s	21.1	2.11 s	21.2		
		170.7		170.7		
OMe					3.17 s	48.3

*^a^* Spectra were measured at 400 MHz; *^b^* Spectra were measured at 100 MHz; *^c^* Spectra were measured at 500 MHz; *^d^* Spectra were measured at 125 MHz.

**Figure 5:**
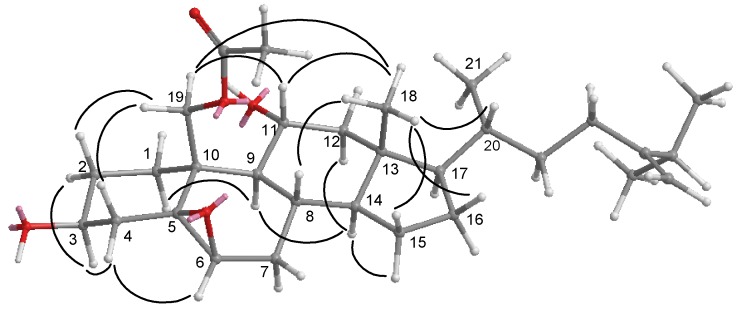
NOESY correlations of compound **2**.

Nebrosteroid O (**2**) was assigned the molecular formula of C_30_H_48_O_5_ based on its HRESIMS and ^13^C NMR data. ^13^C NMR spectra of **2** showed the presence of five methyls, ten sp^3^ methylenes, nine sp^3^ methines, one sp^2^ methylene, three quaternary sp^3^ carbons, and one quaternary sp^2^ carbons. Analysis of the 1D and 2D NMR data (Table 1 and Figure 3) showed that **2** contains one primary acetoxy group [δ_H_ 2.11 (3H, s); δ_C_ 21.2 (q), 170.7 (s)], two secondary hydroxy groups at δ_H_ 3.72 (1H, m), 4.08 (1H, m) and δ_C_ 69.1 (d), 68.9 (d), one trisubstituted epoxy ring [δ_H_ 2.96 (1H, d, *J* = 2.5 Hz); δ_C_ 59.8 (d), 61.3 (s)] and one terminal methylene group [δ_H_ 4.65 (1H, s), 4.72 (1H, s); δ_C_ 156.7 (s), 106.0 (t)]. These spectral data resembled those for the armatinol A [16] except that **2** contained an additional hydroxyl function at C-11. The placement of this moiety was made on the basis of COSY (Figure 3) correlations between H-9, H-11 and H_2_-12. The secondary hydroxyl was deduced to be α oriented based on H_2_-19 and H_3_-18 showing correlations to H-11 in the NOE spectrum (Figure 5).

**Figure 6:**
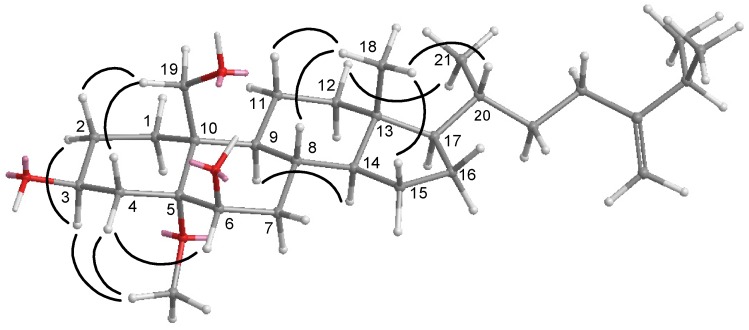
NOESY correlations of compound **3**.

Nebrosteroid P (**3**) had the formula of C_29_H_50_O_4_ as determined by HRESIMS and NMR data thus required five double bond equivalents. The IR spectrum of **3** showed the absorptions for hydroxyl (ν_max_ 3432 cm^−1^) and terminal methylene (ν_max_ 1639, 886 cm^−1^). Its NMR data (Table 2) contain four methyls [δ_H_ 0.72 (3H, s), 0.94 (3H, d, *J* = 6.8 Hz), 1.02 (3H, d, *J* = 6.8 Hz), 1.03 (6H, d, *J* = 6.8 Hz); δ_C_ 12.5, 18.7, 21.9, 22.0)], two oxymethines [δ_H_ 3.88 (1H, m), 3.85 (1H, m); δ_C_ 67.5, 69.4], one oxymethylene [δ_H_ 4.30 (1H, d, *J*= 12.0 Hz), 4.93 (1H, d, *J*= 12.0 Hz); δ_C_ 65.9], a terminal methylene signal [δ_H_ 4.66 (s), 4.71 (s); δ_C_ 105.9 (CH_2_), 156.9 (qC)], and a methoxy group [δ_H_ 3.17 (3H, s); δ_C_ 48.3]. The ^13^C NMR and DEPT spectra of **3** contained ten sp^3^ methylenes, eight sp^3^ methines, three quaternary sp^3^ carbons, one sp^2^ methine, and one quaternary sp^2^ carbon. These spectra data resembled those of armatinol B [16] except that **3** contained a tertiary methoxy instead of a tertiary hydroxyl at C-5. Based on the HMBC correlation from methoxyl protons to C-5 (Figure 3) and NOESY correlations from methoxyl protons to H-3 (Figure 6), nebrosteroid P was elucidated as 5α-methoxy-24-methylenecholestan-3β,6β,19-triol.

Nebrosteroids N–P (**1**–**3**) exhibited cytotoxicity against P-388 cell line with ED_50_ of 0.9, 1.2, and 1.7 μg/mL, respectively (see Table 2). Nebrosteroids N–P (**1**–**3**) were also examined for their antiviral activity towards human cytomegalovirus (HCMV) using a human embryonic lung (HEL) cell line; all compounds were found to be inactive.

**Table 2:** Cytotoxicity andAnti-HCMV Activity of **1**–**3**.

Compounds			ED_50_ (μg/mL)			
	A549	HT-29		P-388	HEL	Anti-HCMV
**1**	6.7	9.5		0.9	23.5	>100
**2**	5.9	5.9		1.2	15.4	>100
**3**	7.2	9.5		1.7	16.1	>100
mithramycin	0.18	0.21		0.15	NT	NT

## 3. Experimental Section

### 3.1. General Experimental Procedures

Optical rotations were determined with a JASCO P1020 digital polarimeter. UV and IR spectra were obtained on JASCO V-650 and JASCO FT/IR-4100 spectrophotometers, respectively. NMR spectra were recorded on a Varian MR 400 NMR spectrometer at 400 MHz for ^1^H and 100 MHz for ^13^C or on a Varian Unity INOVA 500 FT-NMR spectrometer at 500 MHz for ^1^H and 125 MHz for ^13^C, respectively. ^1^H NMR chemical shifts are expressed in δ referring to the solvent peak δ_H_ 7.27 for CDCl_3_, and coupling constants are expressed in Hz. ^13^C NMR chemical shifts are expressed in δ referring to the solvent peak δ_C_ 77.0 for CDCl_3_. MS were recorded by a Bruker APEX II mass spectrometer. Silica gel 60 (Merck, Darmstadt, Germany, 230–400 mesh) and LiChroprep RP-18 (Merck, 40–63 μm) were used for column chromatography. Precoated silica gel plates (Merck, Kieselgel 60 F_254_, 0.25 mm) and precoated RP-18 F_254s_ plates (Merck) were used for thin-layer chromatography (TLC) analysis. High-performance liquid chromatography (HPLC) was carried out using a Hitachi L-7100 pump equipped with a Hitachi L-7400 UV detector at 220 nm together with a semi-preparative reversed-phase column (Merck, Hibar LiChrospher RP-18e, 5 μm, 250 × 25 mm).

### 3.2. Biological Material

The soft coral *N. chabrolii* was collected by hand using scuba at San-Hsian-Tai, Taitong County, Taiwan, in July 2008 at a depth of 12 m and stored in a freezer until extraction. The voucher specimen (SST-22) was identified by Chang-Feng Dai, National Taiwan University and deposited at the Department of Marine Biotechnology and Resources, National Sun Yat-sen University, Taiwan.

### 3.3. Extraction and Isolation

A specimen of soft coral *N. chabrolii* (2.0 kg) was minced and extracted with acetone (3 L × 4) at room temperature. The combined acetone extracts were then partitioned between H_2_O and EtOAc. The resulting EtOAc extract (24.9 g) was subjected to gravity silica gel 60 column chromatography (Si 60 CC) using *n*-hexane and *n*-hexane/EtOAc of increasing polarity, to give 20 fractions. The fraction 13 (0.65 g), eluted with EtOAc, was further subjected to Si 60 CC (EtOAc) to give 7 subfractions. A subfraction 13-6 (299 mg), was separated by a RP-18 flash column (MeOH/H_2_O, 45:55 to 100% MeOH) to give 12 fractions. The subfraction 13-6-11, eluted with MeOH/H_2_O (90:10), was purified by RP-18 HPLC (MeOH/H_2_O, 95:5) to afford **3** (2.4 mg). Likewise, the subfraction 13-7 (177 mg), was separated by a RP-18 flash column (MeOH/H_2_O, 45:55 to 100% MeOH) to give 6 fractions. In turn, a subfraction 13-7-6, eluted with MeOH, was further purified by RP-18 HPLC (MeOH/H_2_O, 90:10) to afford **1** (1.9 mg) and **2** (0.7 mg).

Nebrosteroid N (**1**): White amorphous powder; [α]_D_^25^ +12.8 (*c* 0.1, CHCl_3_); IR (neat) ν_max_ 3393, 2930, 2867, 1738, 1466, 1383, 1237, 1042, 970 cm^−^^1^; ^1^H NMR (CDCl_3_, 400 MHz) and ^13^C NMR (CDCl_3_, 100 MHz) data in Table 1; HRESIMS *m/z* 483.3448 [M + Na]^+^ (calcd for C_29_H_48_O_4_Na, 483.3450).

Nebrosteroid O (**2**): White amorphous powder; [α]_D_^25^−32.2 (*c* 0.1, CHCl_3_); IR (neat) ν_max_ 3394, 2926, 2861, 1737, 1644, 1549, 1461, 1371, 1242, 1044, 889 cm^−^^1^; ^1^H NMR (CDCl_3_, 500 MHz) and ^13^C NMR (CDCl_3_, 125 MHz) data in Table 1; HRESIMS *m/z* 511.3398 [M + Na]^+^ (calcd for C_30_H_48_O_5_Na, 511.3399).

Nebrosteroid P (**3**): White amorphous powder; [α]_D_^25^−44.0 (*c* 0.1, CHCl_3_); IR (neat) ν_max_ 3432, 2950, 2871, 1639, 1458, 1375, 1062, 1028, 886 cm^−^^1^; ^1^H NMR (CDCl_3_, 400 MHz) and ^13^C NMR (CDCl_3_, 100 MHz) data in Table 2; HRESIMS *m/z* 485.3604 [M + Na]^+^ (calcd for C_29_H_50_O_4_Na, 485.3607).

### 3.4. Cytotoxicity Assay

Cytotoxicity was determined on P-388 (mouse lymphocytic leukemia), HT-29 (human colon adenocarcinoma), and A-549 (human lung epithelial carcinoma) tumor cells using a modification of the MTT colorimetric method according to a previously described procedure [28,29]. The provision of the P-388 cell line was supported by J.M. Pezzuto, formerly of the Department of Medicinal Chemistry and Pharmacognosy, University of Illinois at Chicago. HT-29 and A-549 cell lines were purchased from the American Type Culture Collection. To measure the cytotoxic activities of tested compounds, five concentrations with three replications were performed on each cell line. Mithramycin was used as a positive control.

### 3.5. Anti-HCMV Assay

To determine the effects of natural products upon HCMV cytopathic effect (CPE), confluent human embryonic lung (HEL) cells grown in 24-well plates were incubated for 1 h in the presence or absence of various concentrations of tested natural products with three replications. Ganciclovir was used as a positive control. Then, cells were infected with HCMV at an input of 1000 pfu (plaque forming units) per well of a 24-well dish. Antiviral activity was expressed as IC_50_ (50% inhibitory concentration), or compound concentration required to reduce virus induced CPE by 50% after 7 days as compared with the untreated control. To monitor the cell growth upon treating with natural products, an MTT-colorimetric assay was employed [30].

## 4. Conclusion

The first investigation of soft coral *N. chabrolii* collected at San-Hsian-Tai (Taitong County, Taiwan) has led to the isolation of three new 19-oxygenated steroids, nebrosteroids N–P (**1**–**3**). Nebrosteroids N–P (**1**–**3**) exhibited cytotoxicity against P-388 cell line with ED_50_ of 0.9, 1.2, and 1.7 μg/mL, respectively. However, previously isolated cholestene derivatives, nebrosteroids I–K [13] did not show cytotoxicity. In order to rule out the possibility of **3** as an isolation artifact, a solution of **2** was kept at room temperature for three days in the presence of Si-60 or RP-18 gel in MeOH. However, the formation of **3** was not observed.

## Supplementary Files

Supplementary File 1: PDF-Document (PDF, 979 KB)
