# Supplementary material for: New 19-Oxygenated Steroids from the Soft Coral Nephthea chabrolii
Source: Mar Drugs. 2012 Jun 6;10(6):1288–96. doi: 10.3390/md10061288 (PMC3397439; doi:10.3390/md10061288)

Supplementary Materials

Table of Contents

S1. ^{1}H NMR spectrum (400 MHz) of Nebrosteroid N (**1**) in $CDCl_3$. 2

S2. ^{13}C NMR spectrum (100 MHz) of Nebrosteroid N (**1**) in $CDCl_3$. 3

S3. ^{1}H NMR spectrum (500 MHz) of Nebrosteroid O (**2**) in $CDCl_3$. 4

S4. ^{13}C NMR spectrum (125 MHz) of Nebrosteroid O (**2**) in $CDCl_3$. 5

S5. ^{1}H NMR spectrum (400 MHz) of Nebrosteroid P (**3**) in $CDCl_3$. 6

S6. ^{13}C NMR spectrum (100 MHz) of Nebrosteroid P (**3**) in $CDCl_3$. 7

S1. ^{1}H NMR spectrum (400 MHz) of Nebrosteroid N (**1**) in $CDCl_3$.

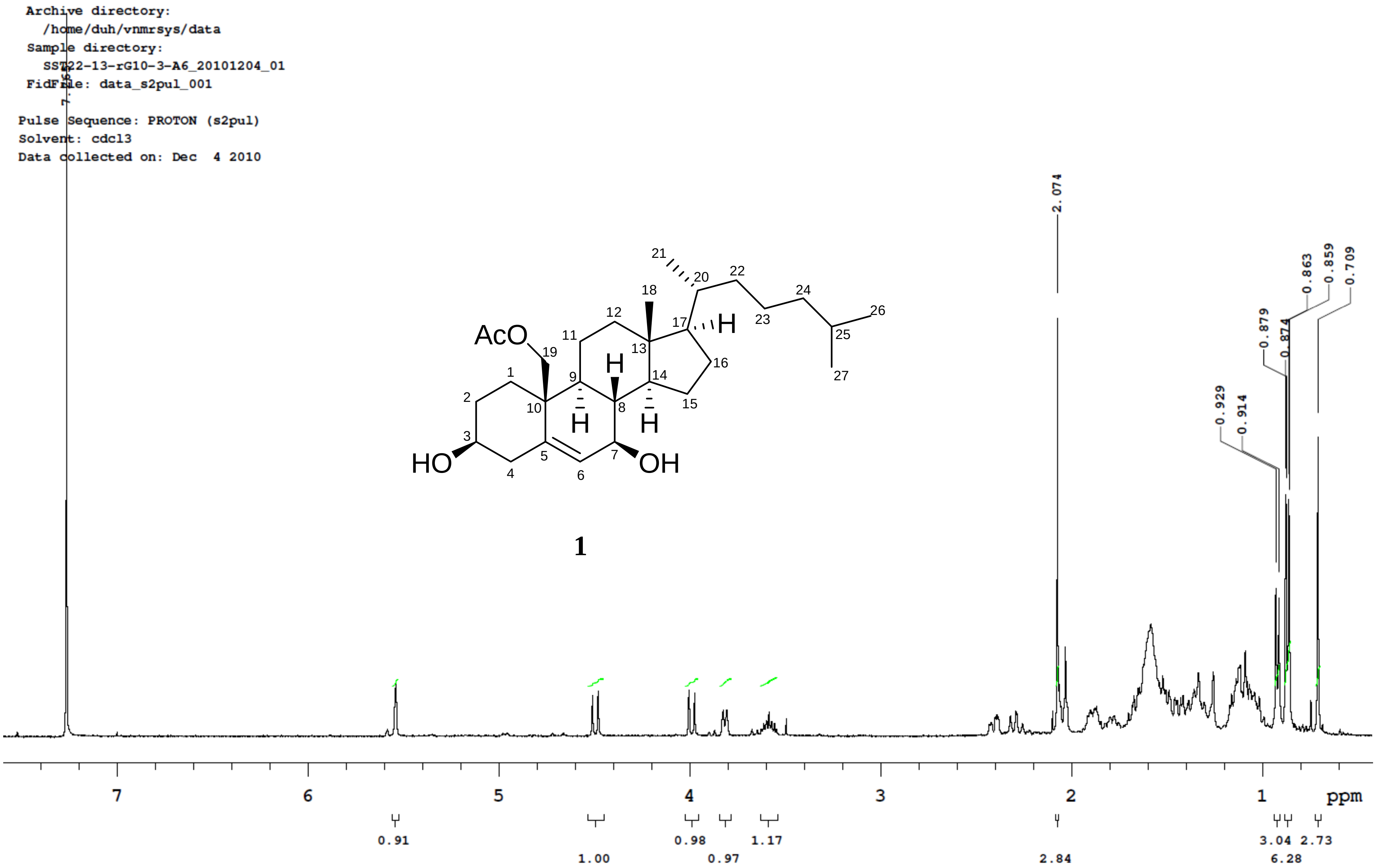

S2. ^{13}C NMR spectrum (100 MHz) of Nebrosteroid N (**1**) in $CDCl_3$.

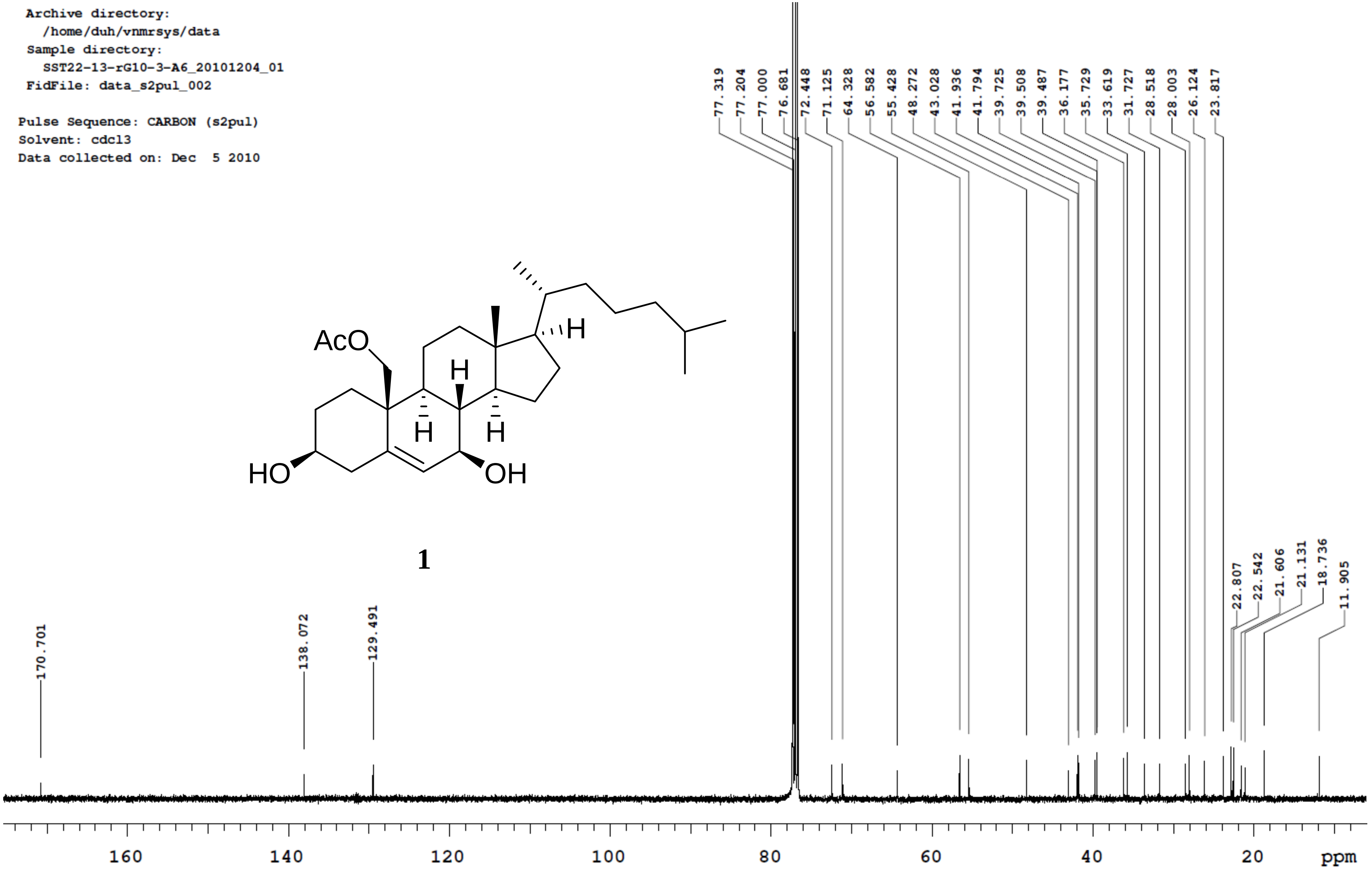

1

S3. ^{1}H NMR spectrum (500 MHz) of Nebrosteroid O (**2**) in $CDCl_3$.

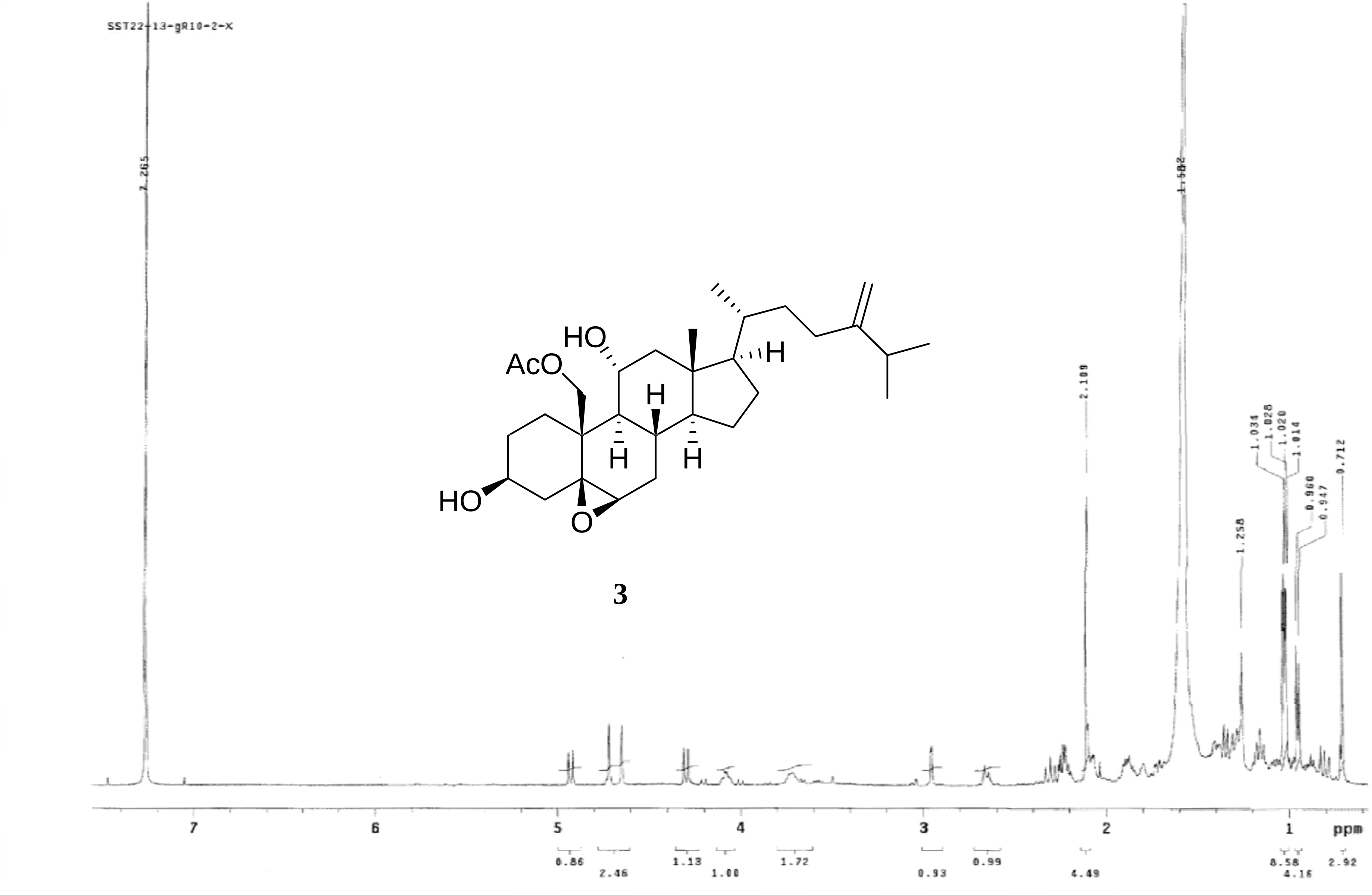

3

S4. ^{13}C NMR spectrum (125 MHz) of Nebrosteroid O (**2**) in $CDCl_3$.

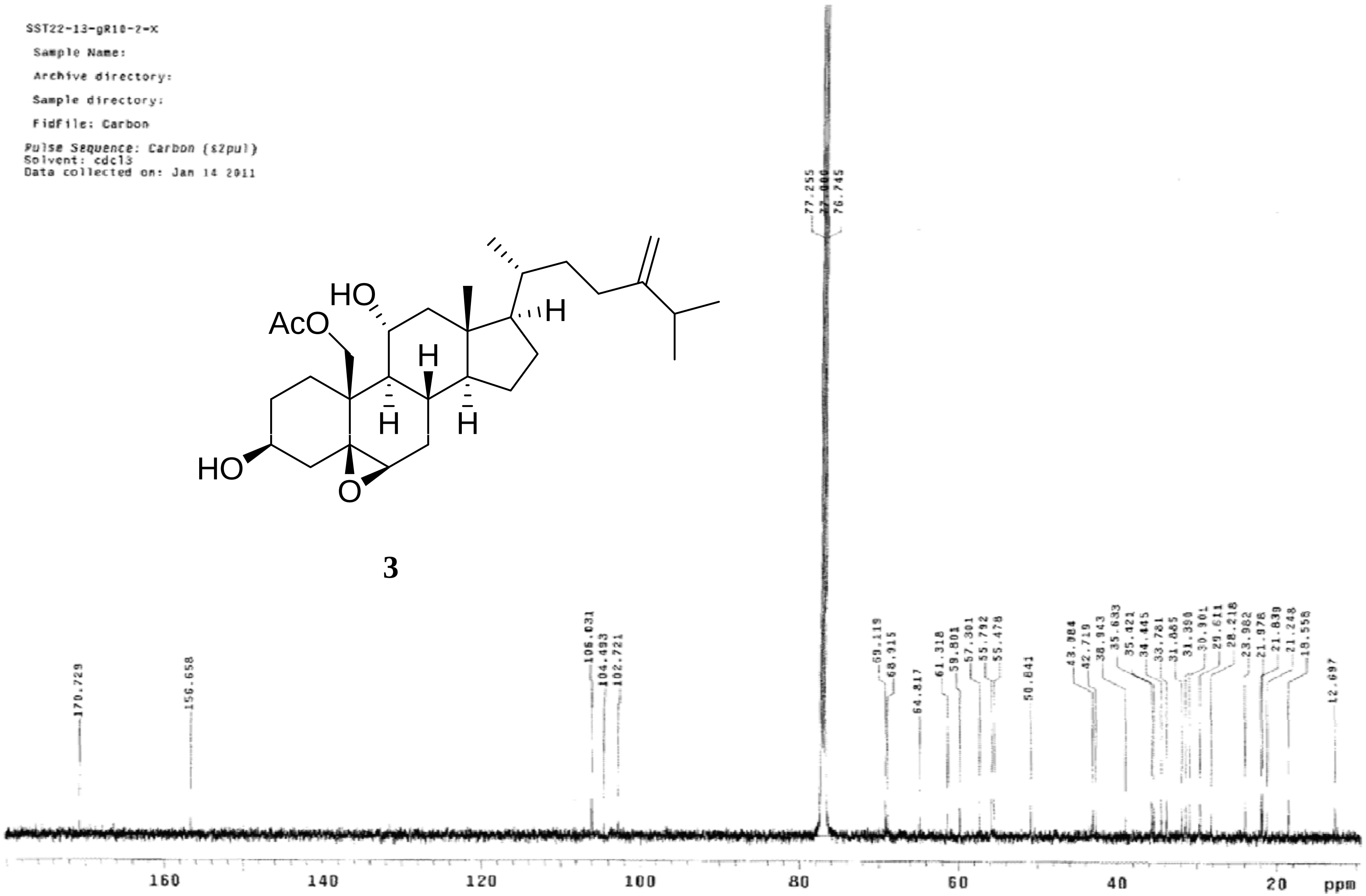

S5. ^{1}H NMR spectrum (400 MHz) of Nebrosteroid P (**3**) in $CDCl_3$.

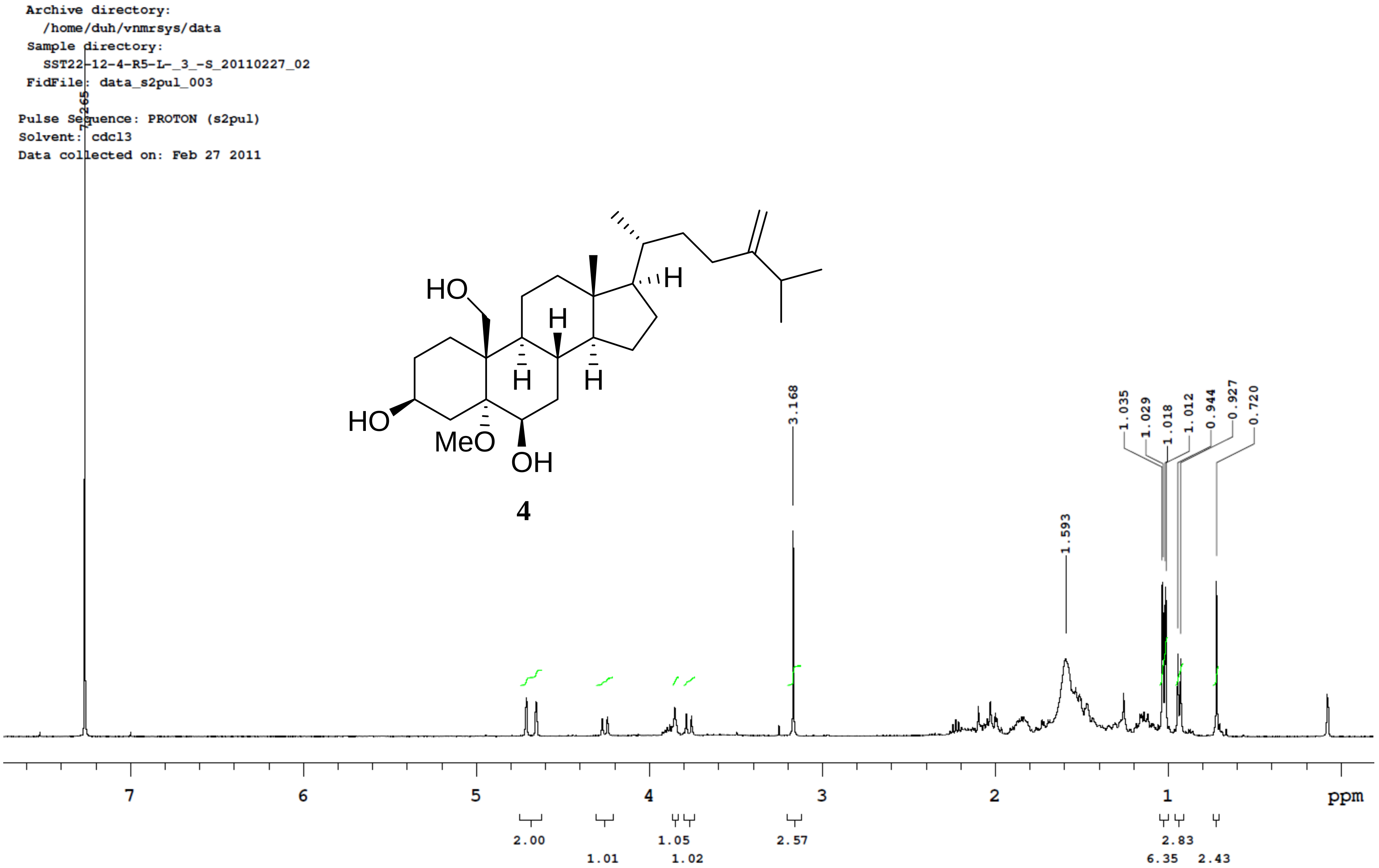

S6. ^{13}C NMR spectrum (100 MHz) of Nebrosteroid P (**3**) in $CDCl_3$.

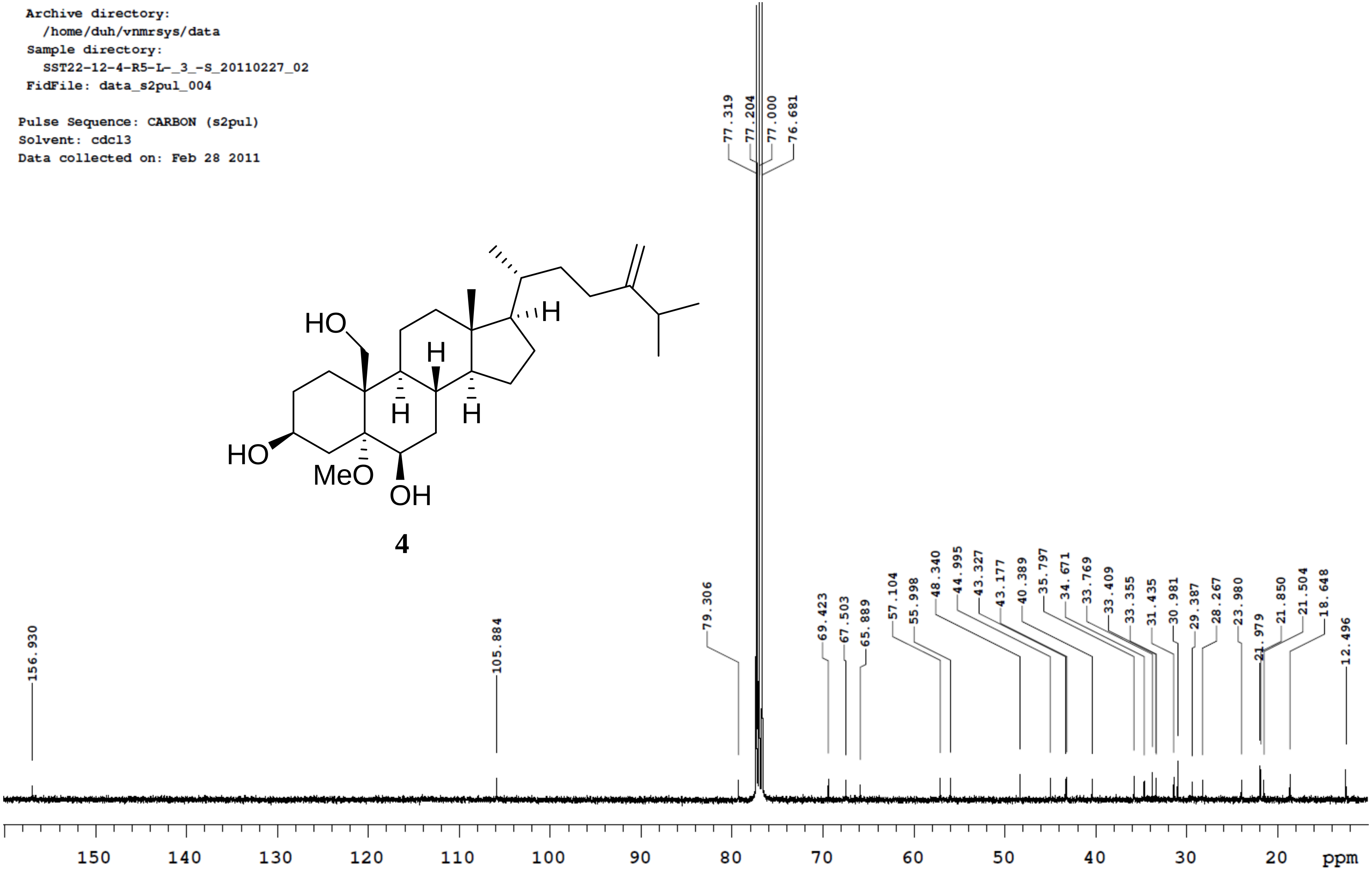

Supplement: Supplementary File 1: — PDF-Document (PDF, 979 KB) [file marinedrugs-10-01288-s001.pdf]
